# Case of Severe Treatment-Resistant Cryptogenic Organizing Pneumonia

**DOI:** 10.15388/Amed.2021.28.2.12

**Published:** 2021-08-26

**Authors:** Domas Grigoravičius, Edvardas Žurauskas, Vygantas Gruslys, Rolandas Zablockis, Edvardas Danila

**Affiliations:** Institute of Clinical Medicine, Faculty of Medicine, Vilnius University, Vilnius, Lithuania; National Center of Pathology, affiliate of Vilnius University Hospital Santaros Klinikos, Vilnius, Lithuania Faculty of Medicine, Vilnius University, Vilnius, Lithuania; Clinic of Chest Diseases, Immunology and Allergology of Faculty of Medicine, Vilnius University, Vilnius, Lithuania Center of Pulmonology and Allergology, Vilnius University Hospital Santaros Klinikos, Vilnius, Lithuania; Clinic of Chest Diseases, Immunology and Allergology of Faculty of Medicine, Vilnius University, Vilnius, Lithuania Center of Pulmonology and Allergology, Vilnius University Hospital Santaros Klinikos, Vilnius, Lithuania; Clinic of Chest Diseases, Immunology and Allergology of Faculty of Medicine, Vilnius University, Vilnius, Lithuania Center of Pulmonology and Allergology, Vilnius University Hospital Santaros Klinikos, Vilnius, Lithuania

**Keywords:** organizing pneumonia, acute respiratory failure, prednisone therapy

## Abstract

Cryptogenic organizing pneumonia is a rare interstitial lung disease with different onset of symptoms, which responds rapidly to glucocorticoid treatment. We present a case of cryptogenic organizing pneumonia which manifested as a progressive 3-year dyspnea that ultimately has led to acute respiratory failure. Moreover, treatment with prednisone for this patient exhibited slow onset of the effect.

## Abstract (lt)

**Gydymui atsparios kriptogeninės organizuojančios pneumonijos atvejis:**

**Santrauka.** Kriptogeninė organizuojanti pneumonija yra reta intersticinė plaučių liga, pasireiškianti įvairiais simptomais, kurie dažniausiai gretai išnyksta gydant gliukokortikoidais. Pristatome kriptogeninės organizuojančios pneumonijos atvejį, pasireiškusį 3 metus progresuojančiu dusuliu ir galiausiai išsivysčiusiu kvėpavimo nepakankamumu. Gydymas prednizolonu šiuo atveju nesukėlė tikėtino greito klinikinio atsako.

**Raktiniai žodžiai: **organizuojanti pneumonija; ūmus kvėpavimo nepakankamumas; gydymas prednizolonu

## Introduction

Organizing pneumonia (OP) is a rare diffuse interstitial lung disease characterized by mesenchymal proliferates (Masson body) in the lung tissue histopathology [1]. There are no specific clinical and radiological findings attributed to this disease [2]. Etiologically OP can be either secondary (secondary to a lung injury such as infection, drug, and pathogen toxicity, gastroesophageal reflux, or radiotherapy and pulmonary lesions of another nature such as vasculitis, lymphoma, lung cancer, hypersensitivity pneumonitis, eosinophilic pneumonia, acute interstitial pneumonia [3]) or cryptogenic [2]. Although the exact prevalence of cryptogenic organizing pneumonia (COP) is unknown, some studies present an estimated incidence of 1 to 3 per 100,000 hospital admissions [4]. Men and women are affected equally [4]. COP typically occurs in patients from 50 to 60 years of age [4], usually nonsmokers [3]. Patients with cryptogenic OP usually present with acute or subacute (days to weeks in duration) respiratory symptoms and rarely develop a progressive disease with severe dyspnea and hypoxemia [4,5]. Moreover, most symptoms are considerably improved shortly after glucocorticoid therapy [4,6]. We introduce an unusual case of the patient with chronic progressive and severe dyspnea leading to acute respiratory failure, and disease itself vaguely responding to glucocorticoid therapy. 

## Case Report

We present a case of a 54 years old women whose main complaint is shortness of breath. Dyspnea started three years ago and progressed from symptoms limited only to physical activity to dyspnea in rest. In 3 years, there were at least two acute viral respiratory infection episodes. The patient is a nonsmoker, has no comorbidities, and no exposition to respiratory toxins or professional hazards. 

**Figure 1.**
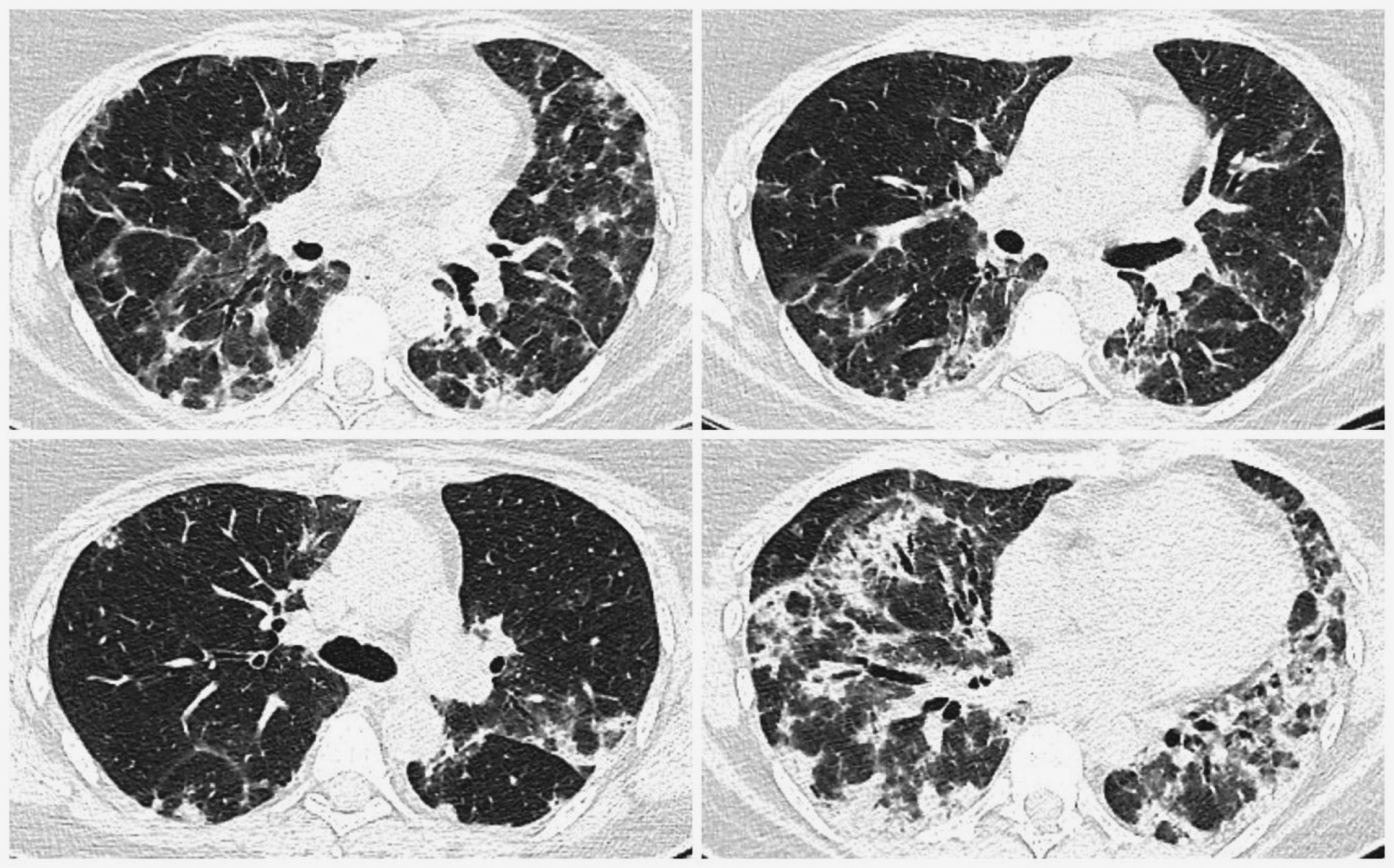
Chest CT (2019.12.06) showing multiple bilateral confluent irregular opacities mainly in the lower lobes, ground-glass appearance, and traction bronchiectasis.

To specify possible causes of these symptoms, a thoracic CT was conducted and showed multiple bilateral confluent irregular foci mainly in the lower lobes, ground-glass appearance, and traction bronchiectasis, all suggesting interstitial lung disease (Figure 1).

**Figure 2.**
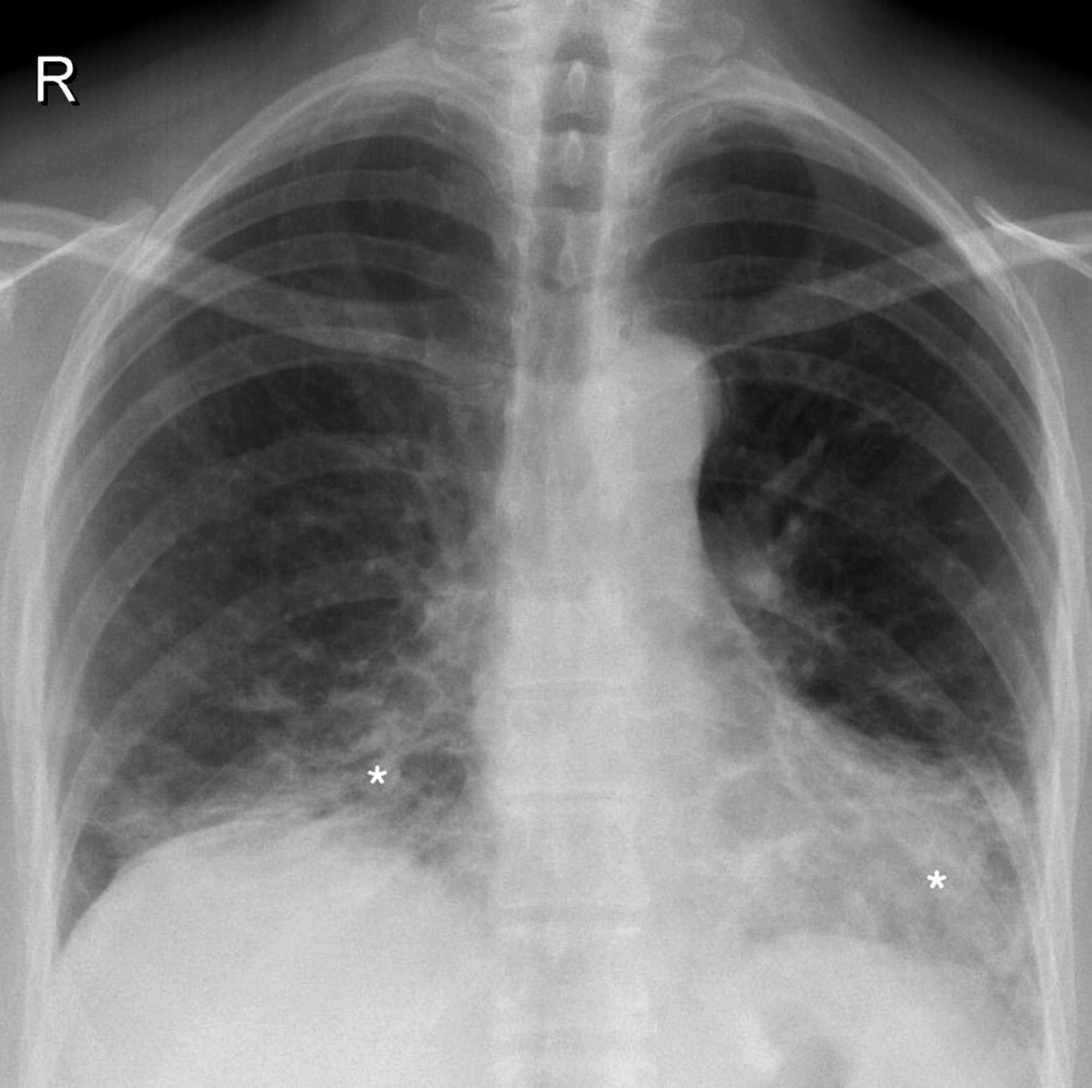
Control chest roentgenogram (2020.02.12) showing bilateral interstitium infiltration (white asterisks) predominantly in the lower segments and focal infiltrative opacities.

Due to progressive dyspnea, further management of the patient was continued in our department. Physical examination revealed a respiratory rate of 19 times per minute in rest and tachypnea during light physical activity (respiratory rate greatly elevated and was immeasurable). In auscultation, no audible rales and normal vesicular breathing were inspected. Peripheral blood oxygen saturation (SpO_2_) on room air was 95% in rest and 82–85% during light physical activity. With oxygen supply, SpO_2_ increased to 90–92%. Arterial blood gas examination showed partial oxygen pressure of 70.7 mmHg. Pulmonary function assessment demonstrated restriction and minor gas diffusion impairment (FVC 3.00 (72%), FEV_1 _2.55 (69%), FEV_1_/FVC 79%, TLC 5.03 (70%), VC 2.98 (72%), RV 1.82 (76%), RV/TLC 79%, DLCO 8.1 (62%)). Control chest roentgenography showed remaining signs of interstitial disease, mostly in the lower lobes (Figure 2).

**Figure 3.**
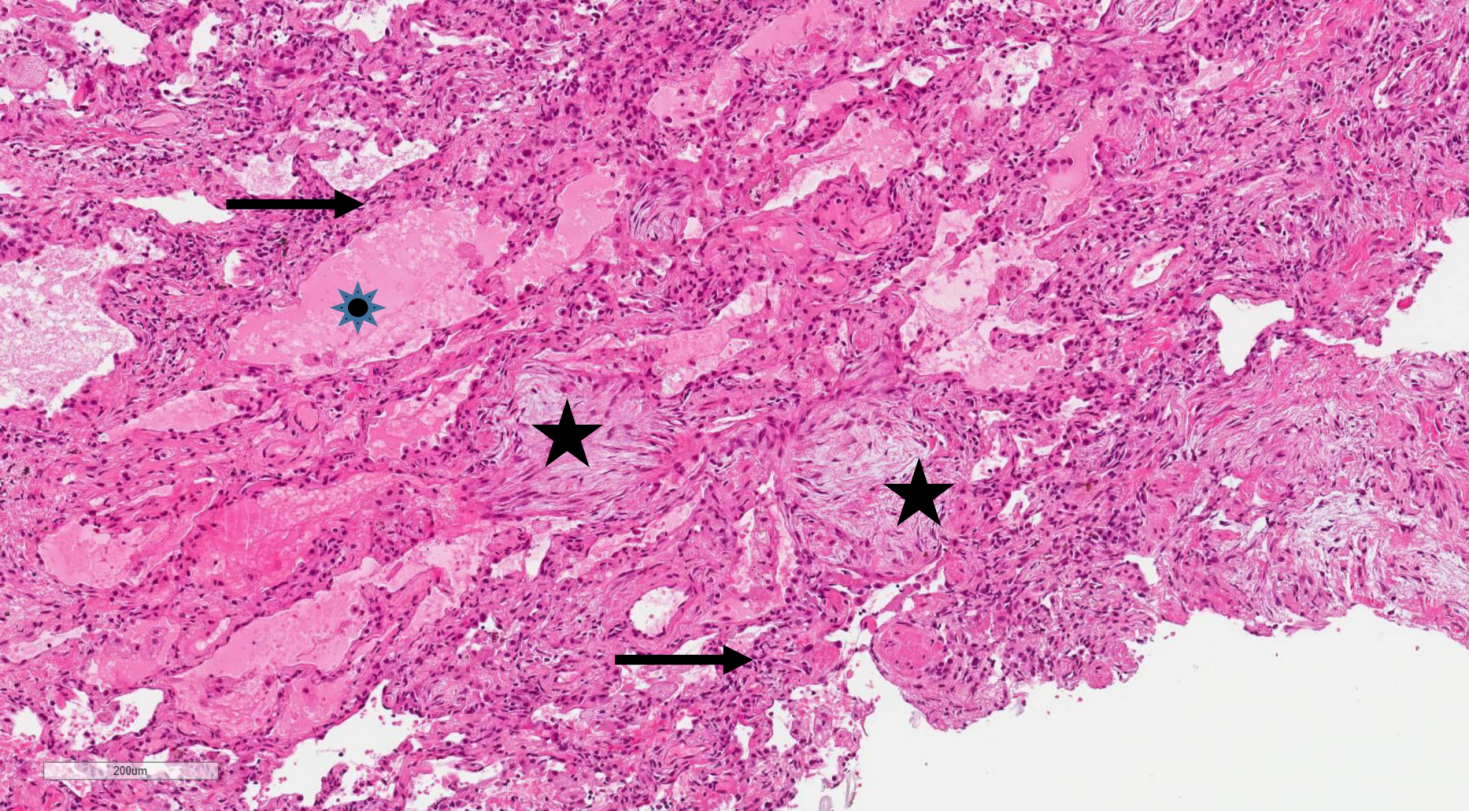
HEx10.Lung biopsy showing mesenchymal proliferates in the alveoli (black stars), thickened alveolar septum with lymphocytes infiltration (black arrows) and edema in the alveolus (blue star).

Further diagnostics included bronchoscopy with lung cryobiopsy and bronchoalveolar lavage (BAL). Histological analysis confirmed interstitial lymphocytic infiltration with organizing pneumonia (Figure 3). BAL microscopic and microbiologic examination appeared without pathological changes: cancerous cells, acid-resistant bacteria, *M. tuberculosis *complex DNA, viruses were not detected. Bacteria, fungi, or *M. tuberculosis *growth was not observed. The patient tested negative for ANA and ANCA.

Based on radiological and histological findings, and absence of etiologic factors patient was diagnosed with cryptogenic organizing pneumonia and was treated with an initial oral dose of 30 mg/d of prednisone for one month and gradually downgraded to 20 mg/d for two months. 

**Figure 4.**
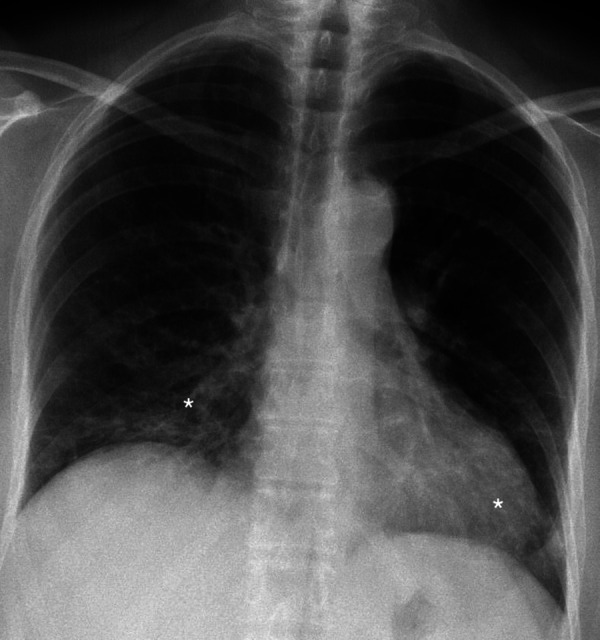
Chest roentgenogram (2020.05.06) after three months of prednisone treatment. Signs of interstitium infiltration (white asterisks) are decreased (improvement more pronounced on the left side) than in the control chest roentgenogram.

After three months patient revisited for treatment control. Subjective patient symptoms were relieved, exercise tolerance increased slightly. Compared with the previous roentgenogram, Chest roentgenography showed minor positive dynamics mainly in the basal segments, although signs of the interstitial disease remain (Figure 4). In pursuance to avoid adverse events, the oral dose of prednisone was gradually reduced to 15 mg/d for three more months.

On the subsequent (after three more months) follow-up, normopnea (respiratory rate of 16 times per minute) in rest and normal lungs auscultation were observed. SpO_2_ in the rest of 96% (without supplemental oxygen) and 92% during physical activity (with supplemental oxygen) was measured. In contrast with the previous test, pulmonary function analysis showed minor positive dynamics, although restriction and gas diffusion impairment remain (FVC 2.97 (80%), FEV_1 _2.53 (80%), FEV_1_/FVC 84%, TLC 5.03 (59%), VC 2.96 (81%), RV 1.83 (32%), RV/TLC 37%, DLCO 8.0 (67%)). Due to the ongoing diminishing of symptoms, prednisone dose was reduced to 10 mg/d and prescribed for three months.

## Discussion

The onset of COP is often no longer than three months with a subacute period of several weeks [7]. The most common symptoms are nonspecific and include malaise, cough, constitutional symptoms, and dyspnea [2,3,8]. The latter usually being chronic, progressive, and mild [9]. However, occasionally COP can be severe in a rapidly progressive form of the disease [9]. Unusually, for our patient COP the course was chronic (3 years), and symptoms progressed to severe dyspnea with acute respiratory failure without apparent reason.

In this case, an essential issue is slow positive dynamics in treating this patient with oral steroids and the need to continue such treatment. Most COP patients are treated with oral glucocorticoids causing rapid symptom relief and radiologic signs improvement [4,10]. However, radiologic signs in some instances can remain for up to several months or not resolve completely at all [11]. In our case, radiologic and symptomatic response to the initial dose of 30 mg/d and after one month shift to 20 mg/day for two more months of prednisone was poor. 

The insufficient clinical effect of glucocorticoids in COP treatment is as high as 40% of all COP cases [12]. In addition, glucocorticoids in nearly one-third of the patients could cause adverse events including gastrointestinal bleeding, arterial hypertension, bone fracture, diabetes mellitus, body-weight increase, and increase mortality rate [6]. Successful treatment of steroid-resistant COP is reported when, in combination with steroids or alone, rituximab [12], azathioprine [4,10], cyclophosphamide, cyclosporine A [4], and mycophenolic acid [13] are applied [14]. 

Furthermore, alternative pharmacological single-drug therapy in patients within normal ranges of pulmonary function parameters are macrolides, such as clarithromycin and azithromycin [6,14]. In comparison with glucocorticoids, macrolides cause fewer side effects and are suitable for patients for whom steroids are contraindicated, or the usage of those is limited [6,14]. Therefore, considering possibilities of alternative treatment and the fact that in a mild course of COP spontaneous regression could be seen, steroids should only be administered in cases of severely affected daily-life and hypoxemia [15], which are present in our patient. 

## Conclusion

It must be considered that COP can present atypically with progressing symptoms, which lead to respiratory failure. Treatment of COP with corticosteroids is the most effective in most cases, although the clinical and radiological effects may not be immediate and needs relatively long-term therapy. Our case report has limitations as the chest computed tomography was not done during a follow-up period.

## References

[ref1] Akyıl FT, Ağca M, Mısırlıoğlu A, Arsev AA, Akyıl M, Sevim T. Organizing Pneumonia as a Histopathological Term. Turk Thorac J. 2017 Jul;18(3):82–7. 29404167 10.5152/TurkThoracJ.2017.16047 PMC5783087

[ref2] Baha A, Yıldırım F, Köktürk N, Galata Z, Akyürek N, Demirci NY, et al. Cryptogenic and Secondary Organizing Pneumonia: Clinical Presentation, Radiological and Laboratory Findings, Treatment, and Prognosis in 56 Cases. Turk Thorac J. 2018 Oct;19(4):201–8. 30322441 10.5152/TurkThoracJ.2018.18008 PMC6196895

[ref3] Baque-Juston M, Pellegrin A, Leroy S, Marquette CH, Padovani B. Organizing pneumonia: What is it? A conceptual approach and pictorial review. Diagnostic and Interventional Imaging. 2014 Sep;95(9):771–7. 24559802 10.1016/j.diii.2014.01.004

[ref4] Chandra D, Maini R, Hershberger DM. Cryptogenic Organizing Pneumonia. StatPearls. Treasure Island (FL): StatPearls Publishing; 2020; Available from: http://www.ncbi.nlm.nih.gov/books/NBK507874/ 29939651

[ref5] Cottin V, Cordier J-F. Cryptogenic Organizing Pneumonia. Semin Respir Crit Care Med. 2012 Oct;33(5):462–75. 23001801 10.1055/s-0032-1325157

[ref6] Radzikowska E, Wiatr E, Langfort R, Bestry I, Skoczylas A, Szczepulska-Wójcik E, et al. Cryptogenic organizing pneumonia—Results of treatment with clarithromycin versus corticosteroids—Observational study. PLoS One. 2017 Sep;12(9). DOI: 10.1371/journal.pone.0184739 PMC5612459 28945804

[ref7] Cordier J-F. Organising pneumonia. Thorax. 2000 Apr;55(4):318–28. 10722773 10.1136/thorax.55.4.318 PMC1745738

[ref8] Bonello RC, Bonello EC, Vassallo C, Bellia EG. Cryptogenic organising pneumonia: an unusual cause of pleuritic chest pain. BMJ Case Reports CP. 2021 Jan;14(1):e238514. 10.1136/bcr-2020-238514 PMC7816905 33462025

[ref9] Cordier J-F. Cryptogenic organising pneumonia. European Respiratory Journal. 2006 Aug;28(2):422–46. 16880372 10.1183/09031936.06.00013505

[ref10] Cottin V, Cordier J-F. Cryptogenic Organizing Pneumonia. Semin Respir Crit Care Med. 2012 Oct;33(5):462–75. 23001801 10.1055/s-0032-1325157

[ref11] Tiralongo F, Palermo M, Distefano G, Vancheri A, Sambataro G, Torrisi SE, et al. Cryptogenic Organizing Pneumonia: Evolution of Morphological Patterns Assessed by HRCT. Diagnostics. 2020 May;10 (5): 262. 10.3390/diagnostics10050262 PMC7277545 32365469

[ref12] Shitenberg D, Fruchter O, Fridel L, Kramer MR. Successful Rituximab Therapy in Steroid-Resistant, Cryptogenic Organizing Pneumonia: A Case Series. RES. 2015;90(2):155–9. 10.1159/000430100 26045243

[ref13] Paul C, Lin-Shaw A, Joseph M, Kwan K, Sergiacomi G, Mura M. Successful Treatment of Steroid-Resistant Fibrosing Organising Pneumonia Causing Respiratory Failure With Mycophenolic Acid. CHEST. 2016 Oct;150(4):277A. 10.1159/000448846 27607231

[ref14] Aslam W, Perez-Guerra F, Jebakumar D, Culver DA, Ghamande S. Acute fibrinous organising pneumonia presenting as a cavitary lung lesion and treatment response to azithromycin. BMJ Case Rep. 2019 Aug;12(8). DOI: 10.1136/bcr-2019-230868 10.1136/bcr-2019-230868 PMC6720639 31439559

[ref15] Kawakami H, Miyabayashi T, Tsubata C, Ota K, Ishida T, Kobayashi O. Spontaneous resolution of thoracic radiation therapy-induced organizing pneumonia: A case series. Respiratory Medicine Case Reports. 2019 Jan;26:180–4. 30705815 10.1016/j.rmcr.2019.01.009 PMC6348731

